# Data Mining of Lung Microbiota in Cystic Fibrosis Patients

**DOI:** 10.1371/journal.pone.0164510

**Published:** 2016-10-14

**Authors:** Jianguo Li, Chunyan Hao, Lili Ren, Yan Xiao, Jianwei Wang, Xuemei Qin

**Affiliations:** 1 Modern Research Center for Traditional Chinese Medicine, Shanxi University, Taiyuan 030006, China; 2 College of Chemical & Biological Engineering, Taiyuan University of Science & Technology, Taiyuan 030021, China; 3 MOH Key Laboratory of System Pathogen Biology and Christophe Mérieux Laboratory, IPB, CAMS-Foundation Mérieux, Institute of Pathogen Biology (IPB), Chinese Academy of Medical Sciences & Peking Union Medical College, Beijing 100730, China; The University of Chicago, UNITED STATES

## Abstract

The major therapeutic strategy used to treat exacerbated cystic fibrosis (CF) is antibiotic treatment. As this approach easily generates antibiotic-resistant strains of opportunistic bacteria, optimized antibiotic therapies are required to effectively control chronic and recurrent bacterial infections in CF patients. A promising future for the proper use of antibiotics is the management of lung microbiota. However, the impact of antibiotic treatments on CF microbiota and vice versa is not fully understood. This study analyzed 718 sputum samples from 18 previous studies to identify differences between CF and uninfected lung microbiota and to evaluate the effects of antibiotic treatments on exacerbated CF microbiota. A reference-based OTU (operational taxonomic unit) picking method was used to combine analyses of data generated using different protocols and platforms. Findings show that CF microbiota had greater richness and lower diversity in the community structure than uninfected control (NIC) microbiota. Specifically, CF microbiota showed higher levels of opportunistic bacteria and dramatically lower levels of commensal bacteria. Antibiotic treatment affected exacerbated CF microbiota notably but only transiently during the treatment period. Limited decrease of the dominant opportunistic bacteria and a dramatic decrease of commensal bacteria were observed during the antibiotic treatment for CF exacerbation. Simultaneously, low abundance opportunistic bacteria were thriving after the antibiotic treatment. The inefficiency of the current antibiotic treatment against major opportunistic bacteria and the detrimental effects on commensal bacteria indicate that the current empiric antibiotic treatment on CF exacerbation should be reevaluated and optimized.

## Introduction

Cystic fibrosis (CF), as an inherited disease of the secretory glands, is caused by defective mutations of the CF transmembrane conductance regulator (CFTR) gene [1–3]. Thickened mucus secretions resulting from the defective CFTR enable the development and persistence of pulmonary bacterial infections [4, 5]. These chronic pulmonary infections, which are mainly caused by opportunistic bacteria such as *Pseudomonas aeruginosa* and *Staphylococcus aureus*, induce intense bronchial neutrophilic inflammation. As a result of chronic polymicrobial infection and inflammation, CF is a devastating life-threatening disease with the majority of mortalities due to respiratory failure [6, 7]. Over the course of CF lung disease two categories have been identified according to disease severity: *exacerbated* and *clinically stable*. Pulmonary exacerbation is the hallmark of CF and is characterized by aggravated pulmonary symptoms and decreased pulmonary function. A clinically stable condition is the stationary phase before or after pulmonary exacerbation.

Unlike the traditional view of a sterile lung, accumulating evidence shows that a microbial community resides in the lung tissue; this is referred to as the lung microbiota [8–11]. Healthy lung microbiota predominately consist of commensal bacteria, including *Streptococcus*, *Veillonella*, *Prevotella*, and *Actinomyces*. [8, 12–14]. Maintenance of a healthy lung microbiota is important for reducing the risk of respiratory tract diseases [12, 15, 16]. Decreased diversity and increased amounts of opportunistic bacteria are notable features of CF lung microbiota compared to uninfected control (NIC) microbiota [17–20]. A substantial reduction in the diversity of airway microbiota paired with stabilized microbial density is associated with CF lung disease progression [8, 17, 21–23].

Chronic and recurrent colonization of opportunistic bacteria causes devastating lung damage and respiratory decline. Intravenous (IV) injection of broad-spectrum antibiotics is the leading therapy for treating pulmonary exacerbation. A typical IV-antibiotic treatment usually requires a duration of two weeks and is inefficient at controlling infections because of the easily generated antibiotic-resistant bacterial strains [24–29]. Thus, effective control mechanisms against opportunistic bacterial infections are needed without generating antibiotic-resistant strains to optimize therapies [22, 30–34].

Management of lung microbiota provides a promising alternative for the treatment of CF with pulmonary exacerbation [8, 10]. Probiotic commensal bacteria of NIC microbiota have substantial and continuous effects on human health and physiological development, including in maturing the immune system and in preventing pathogen invasion [35]. By regulating pathogen activity and enhancing the natural immune system using probiotic supplementary therapies and antibiotic treatments that precisely target opportunistic bacteria, the growth of antibiotic-resistant bacteria can be prevented and the toxicity of these bacteria to NIC microbiota can be reduced [36]. However, several key questions must be answered before microbiota profiling can inform clinical practice. First, how many opportunistic bacteria are there in CF lung microbiota? Second, aside from opportunistic bacteria what are the differences between CF and NIC microbiota? Third, how do antibiotic treatments influence opportunistic and commensal bacteria? Numerous studies have attempted to answer these questions, but little instructive information has been obtained. This is partially due to the small sample size enrolled, which has limited the ability to investigate potential correlations between lung microbiota and CF [12].

This study analyzed the largest number of airway microbiota from CF patients and uninfected controls from published studies to evaluate the differences between uninfected and CF-derived microbiota. The efficacy of the antibiotic treatments in regulating the composition of lung microbiota in CF patients was also evaluated. In general, intravenous antibiotics treatment is the most popular strategies for CF exacerbation, while oral intake of antibiotics is commonly used to control seizures. The antibiotic treatment mentioned in this study refers to the large overdose intravenous therapy for CF exacerbation. Overall, our findings show that CF microbiota were richer and had a lower diversity in the community structure than NIC microbiota The obtained data provide insights necessary to evaluate the current therapeutic strategy.

## Materials and methods

### 1. Definitions

The items used in this study were the same as those defined by Rabin *et al* [8] with some modifications. Briefly, enrolled CF patients were divided into two groups according to disease stages: baseline CF and CF with pulmonary exacerbation (defined as a hospitalization for respiratory symptoms requiring large-dose antibiotic therapy.) The pulmonary exacerbation stage was divided into three subgroups according to the stages of antibiotic administration: BeforeTreat, DuringTreat, and AfterTreat. NIC (uninfected control) was defined an individual without acute or chronic infections such as viral infections, tuberculosis, chronic obstructive pulmonary disease, bronchiectasis, or idiopathic pulmonary fibrosis. Antibiotic therapy or antibiotic treatment in this study specifically referred to the administration of antibiotics necessary to treat CF with pulmonary exacerbation, excluding the use of antibiotics for chronic maintenance.

### 2. Data collection and preprocessing

High-throughput sequencing data of 16s rRNA were collected from public databases (Table 1). Bacterial 16s rRNA genes were PCR amplified and sequenced on different sequencing platforms across the studies (Roche 454 or Illumina Hiseq/Miseq). Primers and targeted regions are listed in Table 1. Raw sequences were filtered and processed using the default parameters of the NGS QC Toolkit version 2.3 [37]. Briefly, for data generated by the Roche 454 (pyrosequencing) platform, sequences that were outside of 200–1000 nucleotides in length, with more than six ambiguous bases, contained homopolymer regions (> 6 bp), had at least two mismatches in the primer, or could not be assigned to a sample by the corresponding barcode were excluded. For data generated by the Illumina (Hiseq and Miseq) platform, paired-end reads were first merged if possible. Reads were then truncated if more than three consecutive low-quality base calls were found, and reads were excluded if ambiguous bases were found after quality trimming.

**Table 1:** Information of the data exploited in this study.

Study/ ACCN	Sample Type	Clinical Status	Sample amount	Treatment before/on/after	Targeted region of 16s rRNA	Sequencing platform	Major findings	Reference
SRP044880	Sputum	Exacerbation	14	6/2/6	V6	454	Intravenous antibiotic therapy often does not profoundly impact bacterial community structure of CF airway microbiome.	[22]
SRP038106	Sputum	Stable	13	-	V4-V6	454	The microbial communities in sputum from pediatric CF patients living together were much more alike than those from pediatric individuals living apart.	[19]
SRP039515	Sputum	Stable	89	-	V1-V2	454	Divergence between microbiota in upper airway compared to lower airway samples, indicating greater differences between communities, was associated with increased sputum neutrophil elastase.	[23]
SRP036061	Sputum	Exacerbation	8	8/0/0	V3-V5	454	Time between Collection and Storage Significantly Influences Bacterial Sequence Composition in Sputum Samples from Cystic Fibrosis Respiratory Infections.	[29]
SRP041296	Sputum	Exacerbation	37	0/37/0	V1-V2	454	CF Pulmonary exacerbation treatment results in variable changes of anaerobic genera suggesting the need for larger studies particularly of patients without traditional CF pathogens.	[53]
SRP025173	Sputum	Stable/Exacerbation	20/43	17/12/14	V4-V6	454	Total and relative abundance of genera at the population level were remarkably stable for individual patients regardless of clinical status.	[51]
SRP039563	Sputum	Exacerbation	8	-	V1-V2	454	As CF control for samples from Chronic Obstructive Pulmonary Diseases.	[54]
SRP040968	Sputum	Exacerbation	8	-	V3-V5	454	Four or more freeze thaw cycles result in a significant distortion of microbiota profiles from CF sputum.	[55]
GAO_CF_ VAMPs	Sputum	Stable/Exacerbation	22/14	-	V4-V6	454	The increased fractional representation of Streptococcus was the strongest predictor of clinically stable CF. Streptococcus may play an important role in increasing the diversity of the CF lung microbiome and promoting patient stability.	[56]
ERP009095	Sputum	Stable	77	-	V3	Miseq	Continuous treatment with antibiotics results in high individuality in CF community composition and lack of correlation to clinical host factors.	[34]
SRP015882	Sputum	Stable/Exacerbation	16/7	0/7/0	V3-V7/V3-V5	454	Diminished microbial diversity is associated with severity of pulmonary inflammation within adult CF cohort.	[48]
Mgm 4603051.3	Sputum	Stable/Exacerbation	15/20	0/7/0	V6	Hiseq	An adult-like lower airways microbiome is established early in life and that throat swabs may be a good surrogate in clinically stable children with CF without chronic Pseudomonas aeruginosa infection in sputum sampling is often not feasible.	[57]
PC01	Sputum	Stable	56	-	V5-V7	454	SI-Seq has a dynamic range of at least five orders of magnitude, can classify .96% of sequences to the genus level, and performs just as well as 454 and paired-end Illumina methods in estimation of standard microbial ecology diversity measurements.	[58]
PC02	Sputum	Stable/Exacerbation	13/49	25/0/24	V1-V2	454	The adult CF lung microbiome is largely stable through periods of exacerbation and antibiotic treatment and short-term compositional changes in the airway microbiota do not account for CF pulmonary exacerbations.	[18]
SRP015882	Sputum	Healthy	9	-	V3-V7	454	Samples from healthy individuals were included as uninfected control	[48]
IPF	BALF	Healthy	28	-	V3-V5	454	Samples from healthy individuals were included as uninfected control	[47]
SRP050998	Sputum	Healthy	73	-	V1-V2	454	Samples from healthy individuals were included as uninfected control	[59]
RVCOPD	Sputum	Healthy	69	-	V3-V5	454	Samples from healthy individuals were included as uninfected control	[45]
PC03	BALF	Healthy	10	-	V3	454	Samples from healthy individuals were included as uninfected control	[60]

### 3. OTU picking

To compare sequences from different regions of the 16s rRNA gene, a reference mapping protocol [38–39] was used to group the sequences into operational taxonomic units (OTUs). Sequences derived from the same bacteria were assumed to match the same reference sequence regardless of the region or length of the targeted 16s rRNA gene. Specifically, the Greengenes database (May 2013 version) [40] of near full-length 16s rRNA was used as the reference database. Close reference OTU picking module in QIIME (version 1.8.0) was used to assign the OTUs [41], and a 97% minimum pairwise nucleotide sequence identity threshold was employed to align sequences in UCLUST [42]. Sequences with identity percentages lower than 97% to any of the reference sequences in the Greengenes database were excluded from further analysis. Moreover, samples with a proportion of assignable sequences less than 85% were excluded. Samples with less than 1000 sequences after quality filtering and OTU assignment were also discarded.

### 4. Alpha diversity measurements

Shared OTUs among different sample groups were plotted in Venn diagrams using Venny software (http://bioinfogp.cnb.csic.es/tools/venny/index.html). Alpha diversity measurements of community richness and diversity were analyzed by estimators of Chao1 and Shannon indices, respectively. Default parameters were applied and boxplots were created using the “Phyloseq” in R package [43]. OTUs of each sample across all studies were then grouped by different taxonomic levels using the workflow script of QIIME according to the 454 overview tutorial (http://qiime.org/tutorials/tutorial.html).

### 5. Beta diversity measurements

To compare the bacterial composition between different groups, unweighted UniFrac analyses of bray-curtis dissimilarity were performed. For this purpose, one thousand sequences were randomly selected from each sample. All analyses were carried out using QIIME.

The 16s rRNA sequencing data and corresponding metadata used in this study are available in publically accessible databases (NCBI SRA [http://www.ncbi.nlm.nih.gov/sra], MG-RAST [https://metagenomics.anl.gov], and VAMPs [https://vamps.mbl.edu/]) with accession numbers listed in Table 1.

### 6. Statistical analyses

Analysis of variance (ANOVA) tests were performed to assess OTUs that significantly differed between sample categories. T-tests were used to determine α-diversity significance. Analysis of similarities (ANOSIM) tests were performed to determine β-diversity significance.

## Results

### Differential community richness and diversity among CF and NIC microbiota

**Fig 1:**
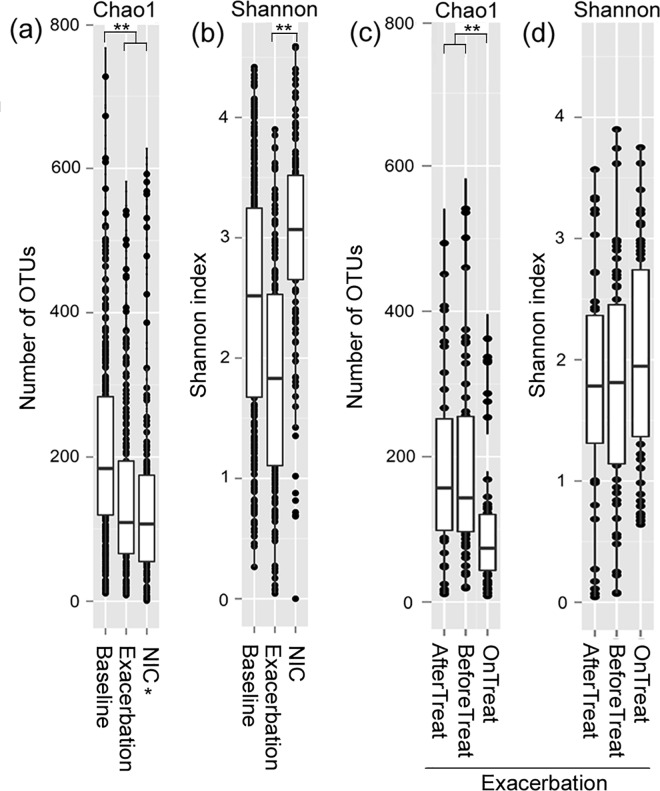
Boxplots for alpha diversity measurements of the microbiota of CF subgroups. Chao1 and Shannon indices were used to determine the richness and diversity of the bacterial community, respectively. Chao1 (a) and Shannon (b) measurements of the microbiota of Uninfected Control (NIC), CF baseline, and Exacerbated CF, Chao1 (c) and Shannon (d) measurements of the microbiota of Before, During and AfterTreatment of exacerbated CF. The upper edge of each box represents the 75% line, the lower edge represents the 25% line, and the inner line of each box represents the 50% line. ** refers to statistically highly significant as *p* < 0.01.

Alpha diversity measurements were used to assess the variation in community structure between the microbiota of CF patients and NICs. Specifically, Chao1 and Shannon indices were employed to analyze community richness and diversity, respectively (Fig 1). These results revealed greater richness of CF Baseline (Fig 1A) and lower diversity of Exacerbated CF (Fig 1B) in CF microbiota than the microbiota of NIC. Comparable levels of bacterial abundance were observed between CF and NIC microbiota, and these levels were remarkably lower than those of CF-Baseline (CF-Base) microbiota (*p* < 0.01, Fig 1A). The microbiota of CF patients with pulmonary exacerbation (CF-Exa) had the lowest community diversity. These results suggest that the microbial community of the CF-Exa microbiota has lower community diversity and richness than NIC microbiota.

**Fig 2:**
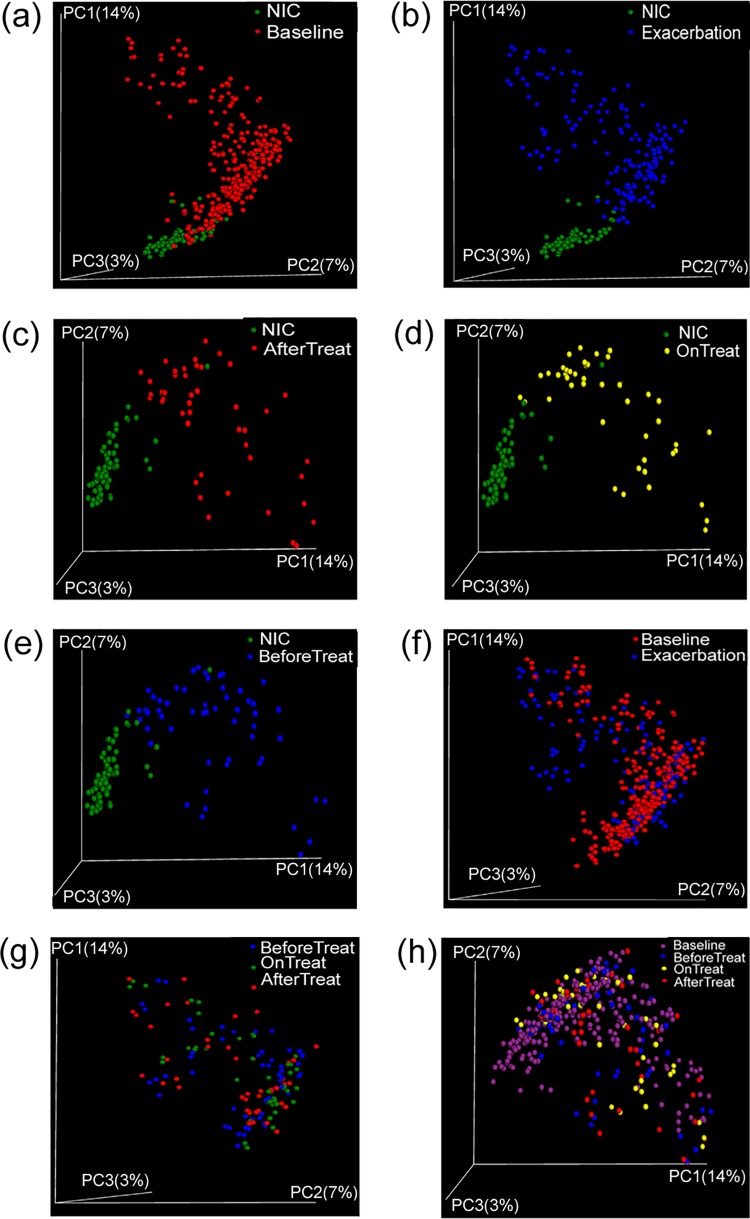
Unweighted UniFrac PCoA plot of healthy and CF microbiota. Distance comparisons were made for microbiota of: (a) Uninfected control (NIC) and CF baseline, (b) NIC and Exacerbated CF, (c) NIC and After Treatment of Exacerbated CF, (d) NIC and On Treatment of Exacerbated CF, (e) NIC and Before Treatment of Exacerbated CF, (f) CF baseline and Exacerbated CF, (g) Before, During and After Treatment of Exacerbated CF, (h) CF baseline and Before, During, After Treatment of Exacerbated CF. Each point represents a sample from the studies listed in Table 1. The color for each sample group can be found on the figure.

As the microbiota of the two CF stages and NICs exhibited divergence in the alpha diversity measurements, beta diversity indices were then calculated to depict this divergence. The unweighted UniFrac-PCoA analysis demonstrated that the microbiota of NICs can be easily distinguished from that of CF patients (Fig 2A and 2B). No marked differences were observed between the microbiota of CF-Base and CF-Exa (Fig 2F). This analysis suggests that there is a differential divergence between the community structure of the NIC microbiota and CF-Base/CF-Exa microbiota, whereas no remarkable divergence is observed between the CF-Base and CF-Exa microbiota.

### CF microbiota was featured with dominant opportunistic bacteria and dramatically decreased commensal bacteria

**Fig 3:**
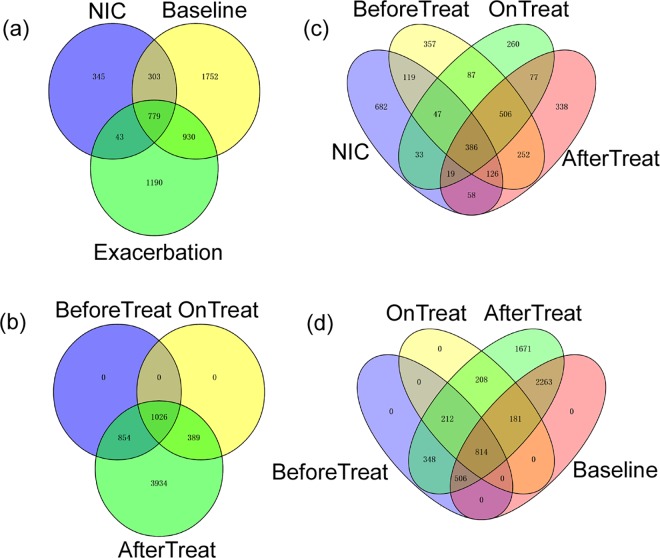
Venn diagrams for the microbiota of healthy individuals and patients in the various CF subgroups. Comparisons were made for: (a) Uninfected control (NIC), CF baseline, and Exacerbated CF; (b) Before, During and After Treatment of Exacerbated CF; (c) NIC and Before, During, After Treatment of Exacerbated CF; (d) CF baseline and Before, During, After Treatment of Exacerbated CF. The number inside each circle represents the amounts of OTUs observed.

Venn diagrams were then generated to analyze the differences in the bacterial composition between the CF and NIC microbiota (Fig 3). The NIC microbiota possessed far fewer bacterial species (1470) than the CF-Base (3764) and the CF-Exa microbiota (2942) (Fig 3A). About 76.5% (1125/1470) of the taxonomies found in the NIC microbiota were also observed in CF microbiota. More specifically, 73.6% (1082/1470) of these taxonomies were present in CF-Base microbiota, 55.9% (822/1470) were present in CF-Exa microbiota, and 53.0% (779/1470) were found in both types of CF microbiota (Fig 3A). About 46.4% (682/1470) of the taxonomies in NIC microbiota were distinct from the treatment-related CF-Exa microbiota (Fig 3C). In contrast, only 29.4% (852/2942) and 28.7% (1082/3764) of the taxonomies identified in CF-Base and CF-Exa microbiota, respectively, were found in NIC microbiota. Furthermore, all of the taxonomies found in CF-Base microbiota were also present in treatment-related CF-Exa microbiota (Fig 3D). These results demonstrate that CF microbiota consist of much higher abundances of taxonomies than NIC microbiota, whereas CF-Exa microbiota harbor the largest number of distinct taxonomies that differ from those of CF-Base and NIC microbiota.

**Fig 4:**
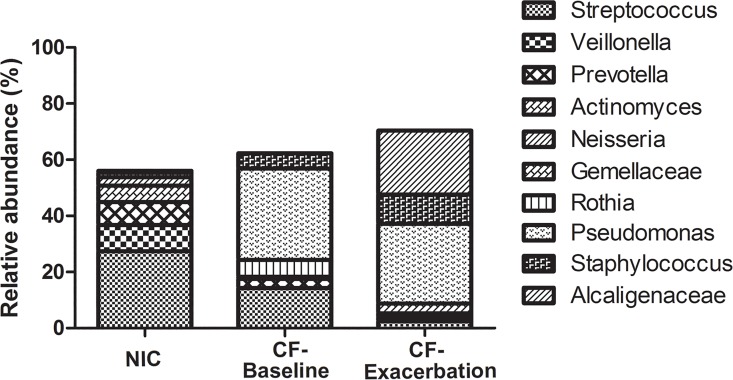
Rank abundance of the dominant bacteria in the microbiota of healthy individuals and patients in the various CF subgroups. Relative abundance of each genus in the three groups (Uninfected control (NIC), CF baseline, and Exacerbated CF) was shown. Only the bacterial genus with relative abundances higher than 3% were shown. Symbol representation of each genus was listed at the right side of the figure.

To probe the differences in taxonomies present in CF and NIC microbiota further, we compared the rank abundance of OTUs at the genus level (Fig 4). Only OTUs with relative abundances larger than 3% were shown. *Pseudomonas* was the major component of CF-Base and CF-Exa microbiota with a relative abundance of approximately 30% (Fig 4); this predominance of *Pseudomonas* was not observed in NIC microbiota (Fig 4). Similarly, *Staphylococcus* and *Alcaligenaceae* were only observed in CF microbiota with high relative abundance (Fig 4) but not in the NIC microbiota (Fig 4). *Streptococcus*, *Veillonella*, and *Prevotella* were the dominant genera in NIC microbiota (Fig 4). These results demonstrate the distinct architectures of CF and NIC microbiota.

### Notable but transient effects of antibiotic treatments were observed in the microbiota of CF patients with pulmonary exacerbation

To evaluate the effects of antibiotic treatment on CF-Exa microbiota, community structures of treatment-related microbiota were compared (Fig 1C and 1D). Consistent with antibiotic treatments, the Exa-DuringTreat microbiota exhibited remarkably reduced community richness (Fig 1C; *p* < 0.01) and slightly increased community diversity (Fig 1D; *p* = 0.24) compared to the microbiota of Exa-BeforeTreat. These findings demonstrate that antibiotic treatment plays an important role on the community structure of the microbiota in patients of CF with pulmonary exacerbation. However, the community richness and diversity of Exa-AfterTreat microbiota returned to levels similar to those of Exa-BeforeTreat microbiota (Fig 1C and 1D), indicating that the effects of antibiotic treatment on the structure of microbiota are transient. These results demonstrate that the notable decrease in community richness in CF-Exa microbiota due to antibiotic treatment was transient, as the architecture of the community returned to Exa-Before after the withdrawal of antibiotics.

To depict the divergence of microbiota caused by antibiotic treatment further, beta diversity indices were analyzed (Fig 2G and 2H). The Unweighted UniFrac-PCoA showed that CF-Exa microbiota could not be discriminated from one another in the community structure (Fig 2G). Moreover, no visible differences were observed between CF-Base microbiota and treatment-related CF-Exa microbiota (Fig 2H). These results demonstrate that the notable effects on community richness caused by antibiotic treatment were transient and did not result in the visible divergence in community structures observed in treatment-related microbiota.

### Antibiotic treatment causes limited effects on the dominant opportunistic bacteria but significant effects on the commensal bacteria

Next, OTUs of CF microbiota were compared to further evaluate the impact of antibiotic treatment on the bacterial composition of CF-Exa microbiota (Fig 3B). The Exa-AfterTreat microbiota covered all of the OTUs of the Exa-BeforeTreat and the Exa -DuringTreat microbiota, 59.9% (3394/5663) of which were distinct from the other two groups. Of the 3394 distinct taxonomies in Exa-AfterTreat microbiota, 1.7% (58/3394) and 66.7% (2263/3394) were shared with NIC (Fig 3C) and CF-Base (Fig 3D) microbiota. The rest (1671/3394, 49.2%) of the Exa-AfterTreat microbiota were unique. These results indicate that the withdrawal of antibiotics in CF patients with pulmonary exacerbation correlated with the proliferation of various taxonomies in CF-Exa microbiota.

To further assess the differences in bacterial composition among CF exacerbation microbiota, OTUs of the microbiota from treatment-related stages of CF with pulmonary exacerbation were compared (Fig 4). Only the OTUs with relative abundances more than 0.5% were shown. Antibiotic treatment dramatically reduced the number of OTUs with high relative abundance (Fig 4). The relative abundances of the *Staphylococcus*, *Gemella*, *Actinomyces*, *Moraxellaceae*, and *Fusobacterium* genera were notably reduced, whereas those of, the *Prevotella* and *Streptococcus* genera were increased, and those of the *Pseudomonas* and *Alcaligenaceae* genera were not affected. After the withdrawal of antibiotics, the richness of the OTUs with high relative abundances was either restored or greater than that of Exa-BeforeTreat microbiota (Fig 4). Specifically, the relative abundances of *Staphylococcus*, *Gemella*, *Actinomyces*, and *Moraxellaceae* were restored (Fig 4). These results demonstrate that antibiotic treatments had no notable effect on the relative abundance of the dominant opportunistic bacteria, but they significantly reduced the relative abundance of commensal bacteria.

### Consistency of the results across studies despite variables

**Fig 5:**
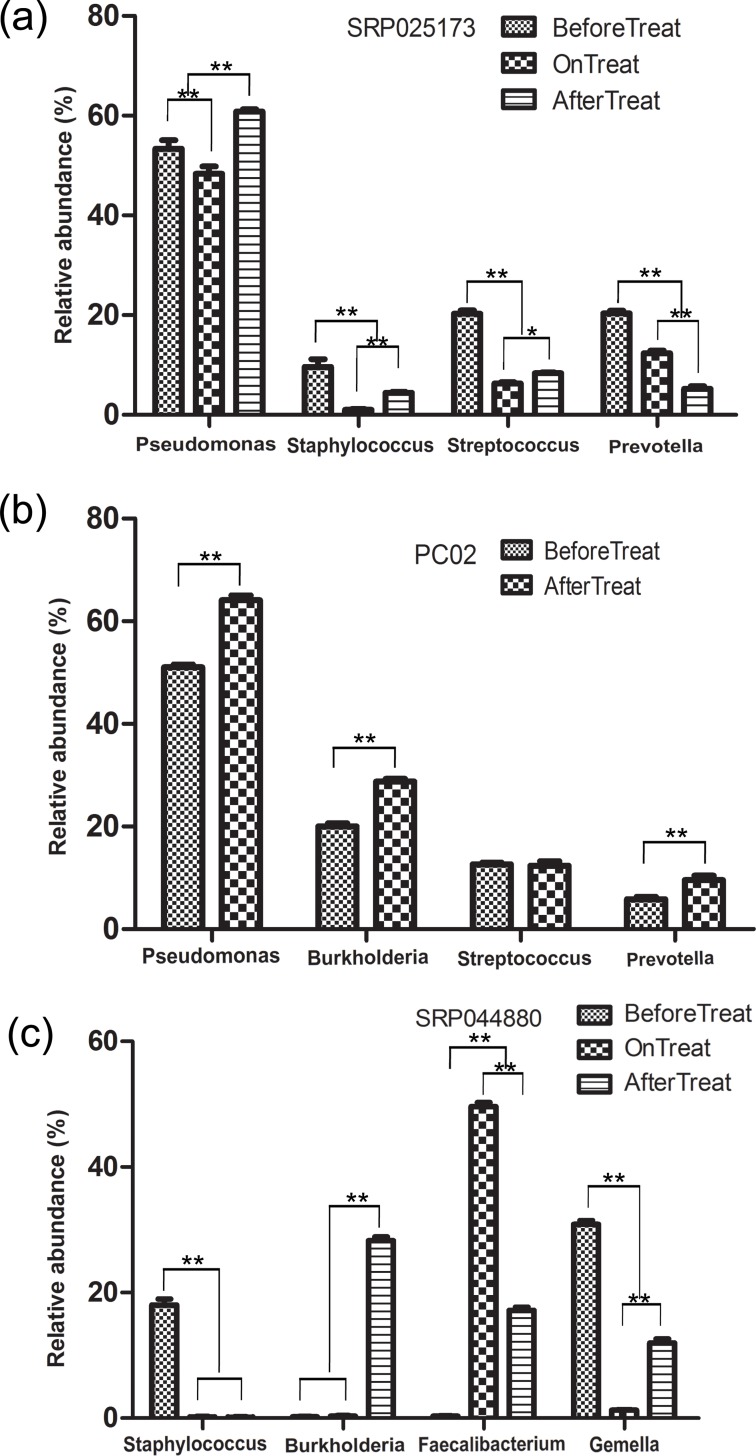
Dynamic variance of the relative abundances for the various bacteria in microbiota of exacerbated CF generated from individual studies. The relative abundances of the top two opportunistic and commensal bacteria are shown. The blank, zebra crossing, and solid column represent the Before, During and After Treatment of Exacerbated CF, respectively. (a) Data from SRP025173, (b) PC02, and (c) SRP044880. Detailed information for these three studies is listed in Table 1. * refers to statistically significant as *p* < 0.05, ** refers to statistically highly significant as *p* < 0.01.

To verify that the results above were not affected by the cross-sectional data enrolled in this study, CF-Exa microbiota from individual studies were analyzed separately using the same close reference OTU picking strategy described above (Fig 5). Analyses of the microbiota data from SRP025173 demonstrated that the relative abundances of the opportunistic bacteria, *Pseudomonas* and *Staphylococcus*, were slightly affected by antibiotic treatments but were restored after the withdrawal of antibiotics, whereas the relative abundances of the commensal bacteria, *Streptococcus* and *Prevotella*, were significantly reduced and were not restored after the withdrawal of antibiotic treatment (Fig 5A). Analyses of the microbiota data from PC02 demonstrated that the relative abundances of the opportunistic bacteria *Pseudomonas* and *Burkholderia* were higher in microbiota after antibiotic treatments compared to those before treatment (Fig 5B). Although the presence of the opportunistic bacteria *Staphylococcus* was diminished following antibiotic treatments, new opportunistic bacteria including *Burkholderia* appeared with higher relative abundance. Moreover, the relative abundances of the commensal bacteria were significantly reduced following treatment (Fig 5C). These results confirm that antibiotic treatments significantly affect the levels of commensal bacteria but do not impact the dominant opportunistic bacteria following the withdrawal of antibiotics.

**Fig 6:**
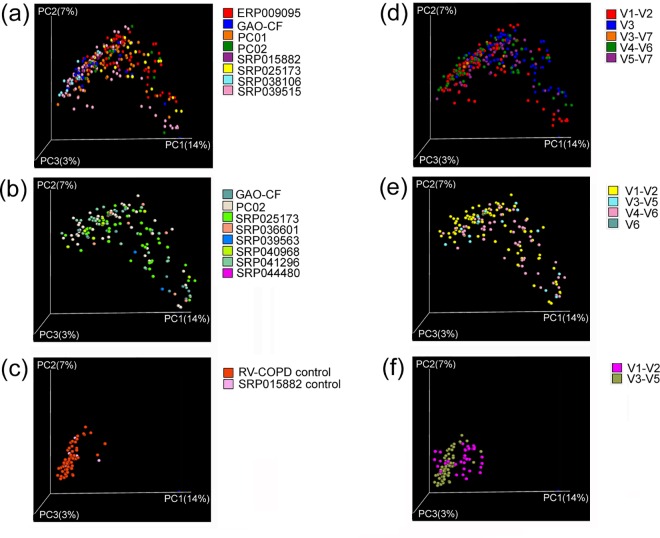
Beta-diversity consistency across studies and 16s rRNA regions. Samples of (a-b) CF baseline, (c-d) Exacerbated CF, and (e-f) Uninfected control (NIC) were compared with queries of studies (a, c, e) and 16s rRNA regions (b, d, f).

To verify the consistency of results across studies despite inherent variables, beta diversity measurements of Clinically Stable, Exacerbated, and NIC samples were compared using query of study assessments of the 16s rRNA region (Fig 6). No significant differences were observed across these studies (Fig 6A) or in the 16s rRNA regions (Fig 6B) in the Clinically Stable (Fig 6A and 6B), Exacerbated (Fig 6C and 6D), and NIC (Fig 6E and 6F) samples. These results highlight the consistency of the results in this and other studies.

## Discussion

This study analyzed a large cohort of lung microbiota samples from CF patients and healthy individuals from cross-sectional studies to compare the community structures and to evaluate the impact of antibiotic treatment on lung microbiota. A sophisticated methodology for handling cross-sectional microbiota studies was used to ensure the reliability of the acquired results [38–39]. Overall our results demonstrate that antibiotic treatments for CF exacerbation have limited effects on opportunistic bacteria but dramatic effects on commensal bacteria. These findings highlight the critical need to reevaluate and optimize the current strategy of antibiotic treatments used to treat CF patients with pulmonary exacerbations.

Healthy microbiota in the airway mucosa is essential to properly shape the immune response. Changing a well-balanced “healthy” microbiota to an unhealthy restricted microbiota renders the airway mucosa increasingly susceptible to pathogens such as *Pseudomonas* and consequent lung injury [44, 45, 46]. Thus, comparing the differences between CF and NIC microbiota to evaluate the dynamic changes in CF lung microbiota is important for understanding the resilience of the polymicrobial ecosystem to intense antibiotic treatments. Unlike previous studies (Table 1), this study enrolled uninfected healthy lung microbiota to serve as a reference for comparison between CF and NIC microbiota. We also evaluated the effects of antibiotic treatments on CF lung microbiota. Consistent with the results of other chronic lung diseases (i.e., chronic obstructive pulmonary disease [16], asthma [12], interstitial pneumonia [47], *etc*.), the structure of the bacterial community in the NIC microbiota was completely different from that found in the CF microbiota. CF microbiota had greater community richness and lower diversity compared to NIC microbiota, indicating that the colonization of opportunistic bacteria had important effects on the community structure of the lung microbiota. These differences were further supported by beta diversity analyses and were consistent with previous reports [48], demonstrating that CF microbiota is distinct from NIC microbiota.

Many studies have long searched for a correlation between certain lung microbes and the severity of CF disease. To date, only the loss of microbial diversity has been associated with severe lung disease in CF [49]; this notion was further supported by the results of the present study. However, the driving force for this decreased microbial diversity remains unclear because there are many complexities involved in the progression of CF disease including lung function, patient age, and antibiotic treatment. Antibiotic treatment, rather than lung function or patient age, was reported to be the primary cause of the decrease in microbiota diversity noted in CF patients who declined from clinically stable to exacerbated states [50]. Nevertheless, indistinguishable bacterial community structures were observed between CF patients deemed as clinically stable and those categorized as exacerbated [18, 51]. However, heavy doses of antibiotics are commonly used to control exacerbated CF states rather than maintain clinically stable states [33]. Thus, the correlation between antibiotic treatments and CF disease progression is weak.

Although intense empiric antibiotic treatment is commonly used to control CF with pulmonary exacerbation, its effects on CF-Exa microbiota must be evaluated to further optimize this strategy [27, 33, 52]. Here, we evaluated healthy and CF clinically stable microbiota as references, which revealed that the most obvious effect of antibiotic treatments on CF-Exa microbiota was the significant reduction in community richness (Fig 1). These results are consistent with previous reports [33, 50]. However, there was no reduction in the opportunistic bacteria targeted by the antibiotics. The relative abundance of *Pseudomonas* was not significantly affected by antibiotic treatment (Fig 4), which is also consistent with previous reports [18, 51], Thus, since *Pseudomonas* is the major opportunistic bacteria found in CF patients with pulmonary exacerbation, more effective antibiotics targeting *Pseudomonas* species are required to control these bacteria. Interestingly, the relative abundance of *Staphylococcus*, another important opportunistic bacteria, was dramatically reduced by antibiotic treatment but these abundance levels returned to their original levels after the withdrawal of antibiotics. This noteworthy phenomenon has also been reported in a previous study [22] and demonstrates the limitations of current antibiotic therapies. These drawbacks highlight the need to reevaluate and optimize current antibiotic treatments for CF patients with pulmonary exacerbation. Because large overdoses of antibiotics have been commonly prescribed for treating CF to ensure a curative effect, it is reasonable to conclude that the varied outcomes of antibiotics should not be the key factor responsible for the differences observed in this study. Furthermore, the concordance of results between individual data and combined data enrolled in this study (Fig 5) also support this speculation. That our results are consistent with previous studies (Fig 5) also suggests that the methods and results of this study are highly reliable.

In summary, this study employed microbiota data from a collection studies to analyze the differences between the microbiota of uninfected healthy individuals and CF patients and to evaluate the impact of antibiotic treatments for CF. CF-Exa microbiota had comparable richness but lower diversity compared to NIC microbiota. Antibiotic treatments for CF patients with pulmonary exacerbation slightly reduced the presence of opportunistic bacteria, but markedly reduced the abundance of commensal bacteria and allowed the proliferation of untargeted opportunistic bacteria. Thus, this study highlights the deleterious influences of antibiotic treatment to microbiota of CF exacerbation, which emphasizes the need to optimize empiric antibiotic treatments.
